# *Ab initio* Molecular Dynamics Simulation Study of Dissociation Electron Attachment to Lactic Acid and Isomer

**DOI:** 10.1038/s41598-019-56019-4

**Published:** 2019-12-20

**Authors:** Ying Zhang, Zhongfeng Xu, Yongtao Zhao, Xiaoan Zhang

**Affiliations:** 1 School of science, Xi′an Jiaotong University, Xi′an, Shaanxi 710049 China; 2 Xianyang Normal University, Xianyang, Shaanxi 712000 China

**Keywords:** Chemical physics, Atomic and molecular collision processes

## Abstract

Dissociation processes of lactic acid and its isomer formed by low-energy dissociation electron attachment (DEA) in the gas phase are investigated by using *ab* initio molecular dynamics (MD) simulations. The *ab* initio MD simulations using an atom-centered density matrix propagation (ADMP) method are carried out to investigate the DEA dissociation process of lactic acid and its isomer. The analysis of the simulated dissociation trajectories of lactic acid and its isomer indicates that the C-C, C-H, and C-O bonds are cleaved within femtoseconds of the simulation time scale in the DEA dissociation process, and the difference in dissociation trajectory depends on the size of the three basis sets. The simulation results enable us to gain insights into the DEA dissociation process of lactic acid and its isomer. In this work, we present a comparative study of the 6-31 + G(d,p), 6-311++G(2d,2p), and Aug-cc-pVDZ basis sets of the DEA dissociation simulation of lactic acid and its isomer. The comparative study results indicate that the 6-311++G(2d,2p) is an excellent basis set for the ADMP trajectory simulation of lactic acid and its isomer in the DEA dissociation process. The natural bond orbital (NBO) analysis is carried out to characterize variation in the charge population and charge transfer accompanied by the C-C, C-H, and C-O bond dissociation processes for lactic acid and its isomer in the ADMP trajectory simulation. ADMP simulation and NBO analysis of the dissociation trajectory is considered an important initial and decisive step in DEA dissociation dynamics for lactic acid and its isomer.

## Introduction

To account for the underlying dynamics in radiation damage with biological systems, a large number of secondary particles, such as low-energy electrons (less than 20 eV), should be considered; the generation of these particles leads to irreversible damage to cellular, DNA nucleobases, organic acids, and amino acids^1–6^. Although low-energy electrons are resonant attachments to biological systems, they may cause dissociation, including processes termed as dissociation electron attachment (DEA), to form neutral molecular fragmentation of biological systems^7–9^. In DEA, which is a commonly known two-step process, low-energy electrons attach to molecules, forming the short-lived transient negative ions (TNIs) via a Franck–Condon transition. The TNIs dissociate from the final products of fragments of neutral molecules (A) and negative ions (*B*^−^). In general, a fragmentation reaction in the DEA process can be written as follows (for example, the target is diatomic molecules):

$${e}^{-}+AB\to {({{\rm{AB}}}^{\ast })}^{-}\to A+{B}^{-}$$

The intensive DEA research on biological systems (e.g., DNA, proteins, and amino acids) focuses on electron attachment mechanism and molecule dissociation dynamics^10–16^; limited studies report the lactic acid dissociation dynamics in the DEA process. Most importantly, lactic acid and its isomer, which are essential products of metabolism, coexist in organisms. The DEA process can produce fragments that affect the process of metabolism, leading to abnormal vital signs. It is therefore not surprising that lactic acid and isomer which low-energy electrons are attached to lactic acid and isomer forming is the target of theoretical work on DEA dissociation process. Consequently, the dissociation dynamics of lactic acid and its isomer on the DEA process in the gas phase must be researched to understand the radiation damage in biological systems.

Theoretical research of the dissociation trajectory is considered an important initial and decisive step in DEA dissociation dynamics for lactic acid and its isomer. Previous theoretical studies using Schwinger multichannel method, single-center expansion quantum scattering calculation method, eigenchannel R-matrix method, and R-matrix method on DEA dissociation dynamics calculations obtained excellent results^15–19^. These studies concentrated on characterizing the dissociation dynamics of electron molecule resonance in physics^20^, but they could not simulate the subsequent dissociation process. Ab initio molecular dynamics (MD) simulations founded on quantum mechanics methods are available and provide insights into the dissociation dynamics of complicated biomolecules. This approach is well suited for the dissociation dynamics of biological systems, such as dialamine conformers^21^. The recent research of *ab* initio MD used an extended Lagrangian MD method, which includes atom-centered Gaussian basis functions and one-particle electronic density matrix propagation^22–25^. This method is termed the atom-centered density matrix propagation (ADMP). To our best knowledge, the ADMP method exhibits attractive features, such as simulation of research systems through accurate treatment of all electrons or through employing pseudopotentials, the capability to employ large time steps by using small values for tonsorial fictitious mass, application of a wide variety of accurate and effective exchange-correlation functionals, and hybrid density and kinetic energy functionals^22,23,26–28^. Based on its attractive features, the ADMP method is used for the first time to explore the dissociation trajectory of lactic acid and its isomer in the DEA process.

In this work, we perform an efficient ADMP method of simulating the dissociation trajectory of lactic acid and its isomer to show the key aspects underlying the DEA process. The remainder of the paper is organized as follows. In Sec. 2, we briefly outline the computational methods, including several calculation parameters relevant to the present study. In Sec. 3, the results of ADMP of lactic acid and isomer are discussed, and electron transfer in the dissociation process of lactic acid and its isomer is analyzed. Finally, we draw our conclusions and lay out directions for future research in Sec. 4.

## Computational Methods

All calculations presented in this work are performed using the Gaussian 09 software package, and the results are visualized through GaussView5^29^. The ground state geometry of lactic acid and isomer are optimized by employing the Becke3-parameter Lee-Yang-Parr (B3LYP) functional of the density functional theory^30^ coupled with the 6-31 + G(d,p), 6-311++G(2d,2p), and Aug-cc-pVDZ basis sets. This level of theory, which is highly efficient and has become one of the most preferred theoretical methods of geometry optimization and DEA dissociation process in complicated biomolecules systems, successfully predicts the experimentally observed dissociation process^21^. Vibrational frequency calculations are performed to verify that the optimized lactic acid and its isomer correspond to a local minimum at the B3LYP theory level. The vibrational frequencies are calculated at the levels of the B3LYP theory and 6-31 + G(d,p), 6-311++G(2d,2p), and Aug-cc-pVDZ basis sets using the optimized geometries. No symmetry restrictions are applied for geometry optimization and vibrational frequency calculations.

The ADMP method of ab initio MD simulation is used for the classical dissociation trajectory calculation of lactic acid and its isomer; the method is performed by employing the B3LYP functional of density functional theory and 6-31 + G(d,p), 6-311++G(2d,2p), and Aug-cc-pVDZ basis sets. The structural propagation of ADMP simulation is initiated at the Franck–Condon region of the DEA process, with vertical electron attachment to the neutral target of optimized lactic acid and its isomer. The ADMP simulation is carried out to 500 femtoseconds (fs) with a time interval 0.1 fs and is sampled with the initial internal energy 1.0 eV. A total simulation of 5000 steps is run for each trajectory. In our ADMP calculations, no restriction is applied for the dissociation trajectory calculations, so all the dissociations cannot be presented in a finite number of trajectories. The Gibbs free energy for lactic acid and its isomer dissociation processes are calculated at the levels of the B3LYP theory and 6-31 + G(d,p), 6-311++G(2d,2p), and Aug-cc-pVDZ basis sets in the gas phase. Natural bond orbital (NBO) analyses, which provide insights into the DEA dissociation process, are run using the optimized lactic acid and its isomer in this work and at the B3LYP level of density functional theory in conjunction with the 6-31 + G(d,p), 6-311++G(2d,2p), and Aug-cc-pVDZ basis sets.

## Results and Discussion

### Optimization geometries of lactic acid and its isomer

**Figure 1:**
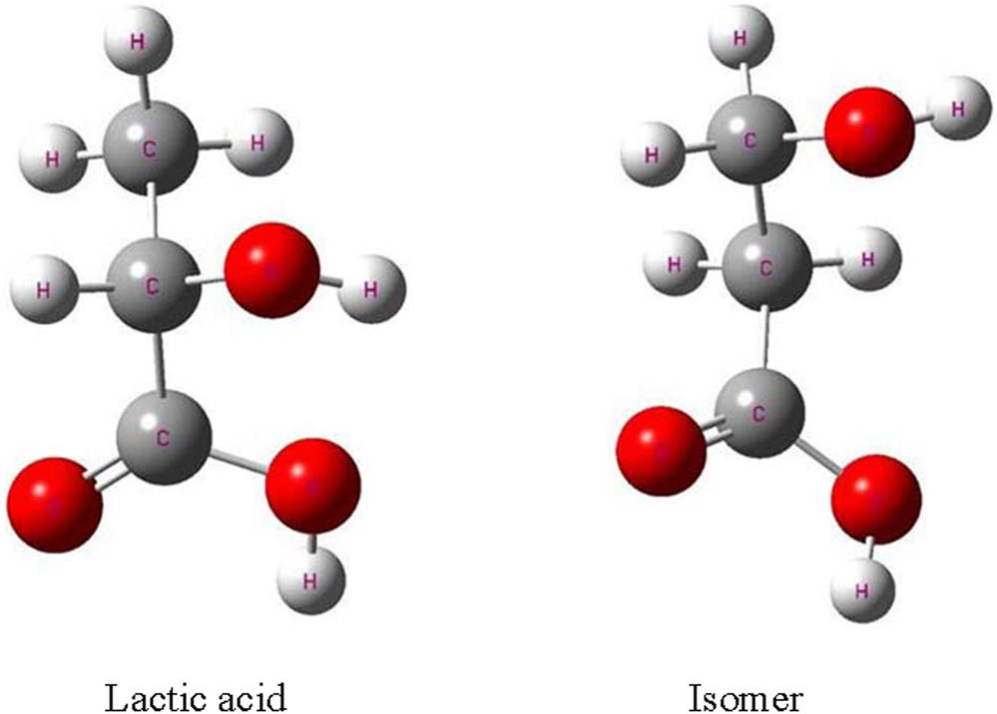
Optimized geometries of ground state lactic acid and its isomer.

Geometry optimization of lactic acid and isomer is a crucial step in the theoretical research of the ADMP dynamics trajectory simulation. The ground state of geometries lactic acid and its isomer are optimized using the B3LYP theory with the 6-31 + G(d,p), 6-311++G(2d,2p), and Aug-cc-pVDZ basis sets. Figure 1 presents the optimized geometries, and Table 1 lists our optimized geometrical parameter results, including bond lengths, angles, and dipole moments, for the ground state lactic acid and its isomer. Analyzing the results of the C-C bond length of the lactic acid and its isomer, the values of the 6-31 + G(d,p) basis set are 1.529 Å and 1.528 Å, respectively. These numbers differ from the bond length observed for the 6-311++G(2d,2p) and Aug-cc-pVDZ basis sets, in which the C-C bond length are 1.527 Å and 1.526 Å; and 1.524 Å and 1.525 Å, respectively. The optimized C-H bond length of lactic acid and its isomer are 1.093 Å and 1.095 Å for the 6-311++(2d,2p) basis set and 1.094 Å and 1.093 Å for the Aug-cc-pVDZ basis set, respectively, and they are slightly shorter than the results obtained for the 6-31 + (d,p) basis set. Notably, the cases of the optimized C-O and O-H bonds with the three basis sets for lactic acid and isomer are same as that for the other optimized bonds. Similar observations can be obtained concerning the optimized bond angle. The6-31 + G(d,p) basis set yields the highest optimized bond angle results among the three basis sets. The 6-31 + G(d,p) basis set, which yield the least values in the three basis sets, elongats bond lengths in geometry optimization for lactic acid and its isomer due to the inclusion of diffuse and polarization functions. Lastly, the Aug-cc-pVDZ basis set results from geometry optimizations are remarkably similar to those obtained for the 6-311++G(2d,2p) basis set. The dipole moment µ in Table 1 displays that the values are larger than 2.8 D with the three different basis sets. According to previous research^21,31^, a large dipole moment indicates the formation of valence-bound anion and the existence of strong electrostatic dipole fields, benefitting the capture of low-energy electron in the DEA process. However, these dipole moments perform poorly in the dissociation trajectory simulation. The 6-31 + G(d,p), 6-311++G(2d,2p), and Aug-cc-pVDZ basis sets are employed to eliminate interferences in dipole-bound interaction for the ADMP dynamics trajectory simulation.

**Table 1:** Optimized geometrical parameters for the ground state lactic acid and its isomer. Bond lengths are measured in Angstrom, whereas angles and dipole moments are in degrees and Debye, respectively.

	6-31 + G (d,p)	Lactic acid	Aug-cc-pVDZ	6-31 + G (d,p)	Isomer	Aug-cc- pVDZ
		6-311++G (2d,2p)			6-311++G (2d,2p)	
R(C-C)	1.529	1.527	1.526	1.528	1.524	1.525
R(C-H)	1.099	1.093	1.094	1.098	1.095	1.093
R(O-H)	0.976	0.967	0.970	0.978	0.968	0.969
R(C-O)	1.450	1.441	1.437	1.430	1.424	1.424
R(C=O)	1.273	1.208	1.220	1.269	1.212	1.215
A(CCC)	112.974	112.610	112.556	112.879	112.723	112.625
A(CCH)	109.787	109.374	109.339	108.427	108.721	108.772
A(CCO)	124.354	124.981	124.635	125.426	124.610	124.676
A(OCH)	106.465	106.167	106.093	106.208	106.351	106.649
A(OCO)	125.868	124.866	124.470	123.881	122.884	122.857
A(HCH)	108.812	108.624	108.643	107.908	107.409	107.340
A(HOC)	111.592	110.634	110.626	108.873	108.18	108.061
μ	3.970	3.624	3.586	3.929	3.664	3.427

The harmonic vibrational frequencies of lactic acid and its isomer, as reported in Tables 2 and 3, are calculated at the B3LYP level for the 6-31 + G (d,p), 6-311++G(2d,2p), and Aug-cc-pVDZ basis sets. Lactic acid and its isomer exhbit minima corresponding to their potential energy surfaces, where magnitudes of all the predicted vibrational frequencies are real positive values. No imaginary frequencies are detected for lactic acid and its isomer. As shown in Tables 2 and 3, the C-C bond stretching vibrational frequencies of lactic acid and its isomer with the 6-311++G(2d,2p) and Aug-cc-pVDZ basis sets are 787.69, 787.75, 811.66, and 811.54 cm^−1^, respectively. Similar results show that the C-H bond stretching vibrational frequencies of lactic acid and its isomer with the 6-311++G(2d,2p) and Aug-cc-pVDZ basis sets are also predicted to be of low values at 1822.21 and 1821.77; and 1791.09, and 1790.66 cm^−1^, respectively. Table 2, which shows the O-H bond wagging vibrational frequencies of lactic acid, provides small different values from the 6-31 + G(d,p) and 6-311 + G(2d,2p) basis sets. The difference implies that the O-H bond wagging vibrations are mainly effected by the hydroxymethyl group stretching vibrations. For lactic acid and its isomer, the IR intensities of the O-H bond wagging vibration results obtained from the 6-311++G(2d,2p) basis set are approximately 370.23 and 326.72 km mol^−1^, respectively, which are the highest and may serve as experimental fingerprints for identifying lactic acid and its isomer. Compared with the given harmonic vibrational frequencies results of lactic acid and its isomer with the three basis sets, the results by the 6-31 + G(d,p) basis set exhibit distinct differences from the findings obtained by the 6-311++G(2d,2p) and Aug-cc-pVDZ basis set. Consequently, the distinct discrepancy obtained using the three basis sets at the B3LYP theory level indicates that the DEA dissociation trajectories of lactic acid and its isomer should be different. The C-O bond vibrational mode of lactic acid is in stretching vibrational mode, whereas that of the isomer is in wagging vibration mode. This finding implies that the DEA dissociation processes of lactic acid and isomer should also differ.

**Table 2:** Frequencies (cm^−1^) and infrared (IR) intensities (km mol^−1^) of the C-C, C-H, and C-O bonds in stretching vibrational mode and the O-H bond wagging vibrational mode for lactic acid.

	C-C		C-O		C-H		O-H	
	Freq.	IR Inten^a^	Freq.	IR Inten.	Freq.	IR Inten.	Freq.	IR Inten
6-31 + G(d,p)	787.47	14.85	1348.79	51.93	1825.59	299.40	1178.43	369.33
6-311++G(2d,2p)	787.69	15.88.	1348.85	56.82	1822.21	311.55	1178.96	370.23
Aug-cc-pVDZ	787.75	15.42	1348.92	56.68	1821.77	308.83	1178.47	369.68

**Table 3:** Frequencies (cm^−1^) and IR intensities (km mol^−1^) of the C-C and C-H bond stretching vibration modes and the C-O and O-H wagging vibrational modes for the isomer.

	C-C		C-O		C-H		O-H	
	Freq.	IR Inten^a^.	Freq.	IR Inten.	Freq.	IR Inten.	Freq.	IR Inten.
6-31 + G(d,p)	812.65	13.14	1410.97	117.71	1795.84	302.73	1152.53	303.11
6-311++G(2d,2p)	811.66	12.68	1404.89	103.96	1791.09	307.55	1147.01	326.72
Aug-cc-pVDZ	811.54	12.51	1402.92	101.60	1790.66	304.94	1144.28	313.86

### ADMP simulation of lactic acid and isomer dissociation process

**Figure 2:**
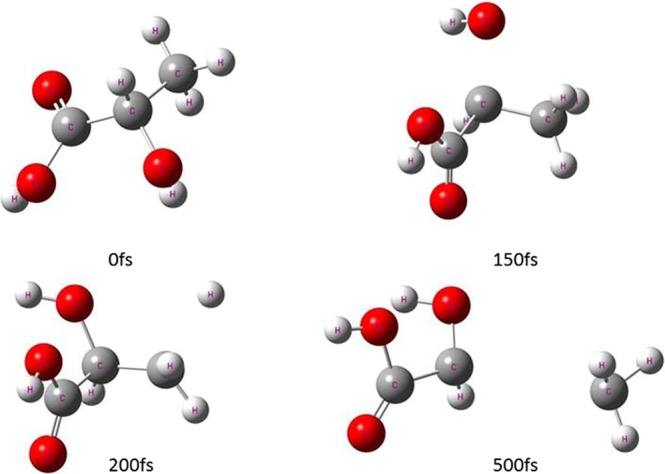
Typical snapshots after vertical electron attachment to lactic acid in the 500 fs ADMP dynamics simulation. Colors in graphical representations correspond to atom types as follows, red: oxygen; white: hydrogen; and gray: carbon.

**Figure 3:**
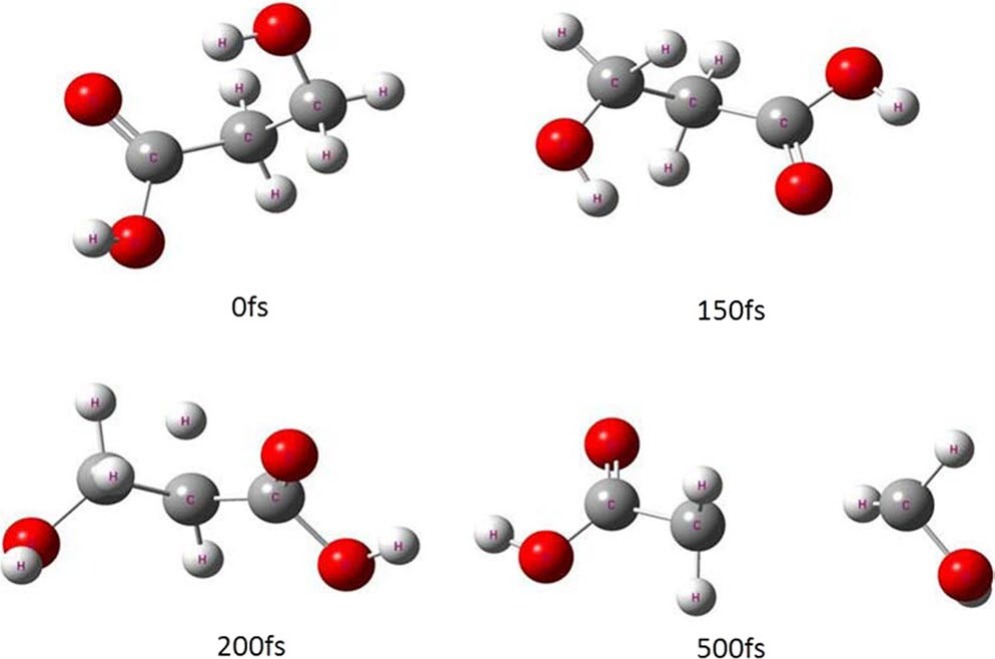
Typical snapshots after vertical electron attachment to the isomer in the 500 fs ADMP dynamics simulation. Colors in graphical representations correspond to atom types as follows, red: oxygen; white: hydrogen; and gray: carbon.

**Figure 4:**
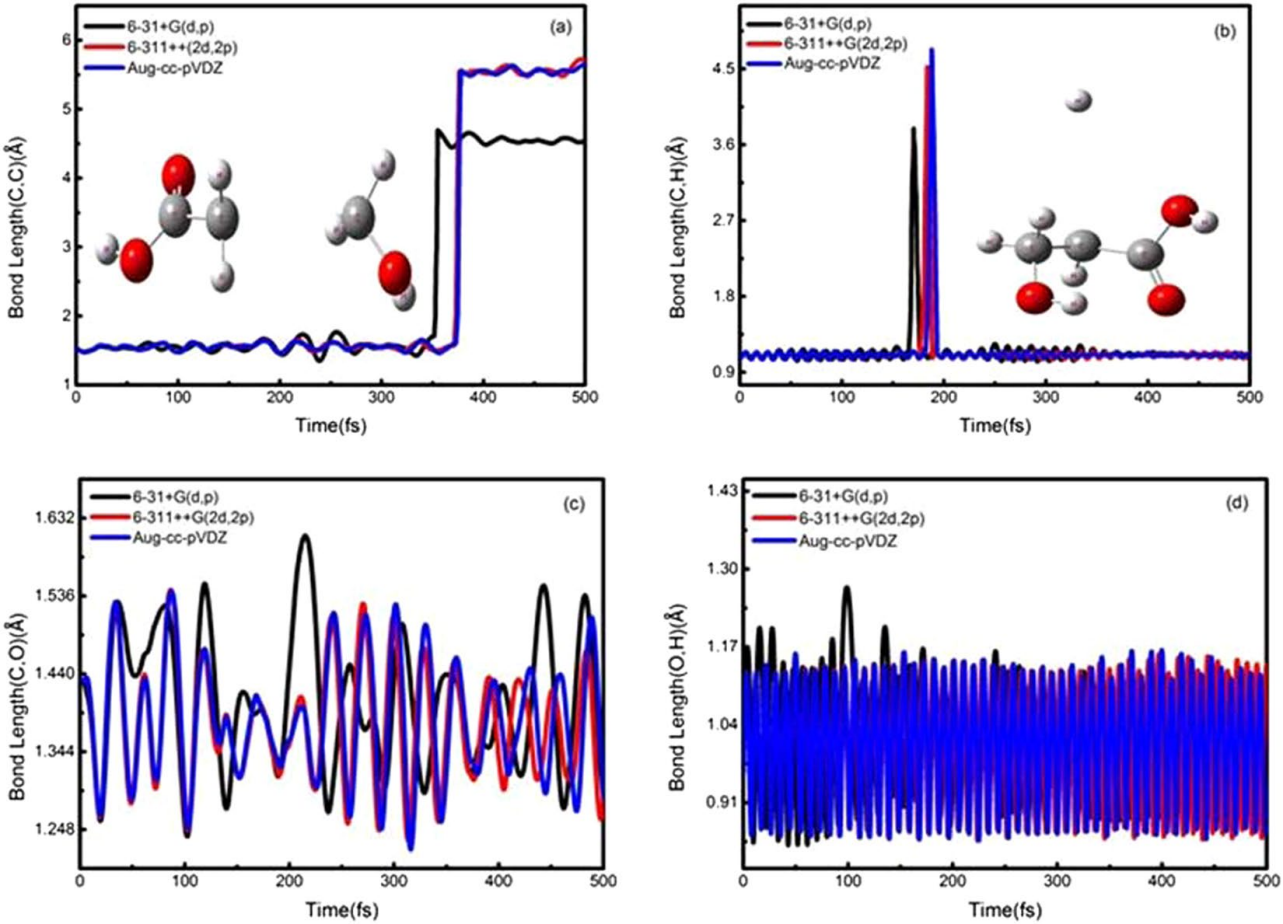
Selected bond distances as a function of evolution of time in the ADMP simulation process for lactic acid: (**a**) C-C bond, (**b**) C-H bond, (**c**) C-O bond, and (**d**) O-H bond. The different colors of trajectories represent the calculation results of different basis sets.

**Figure 5:**
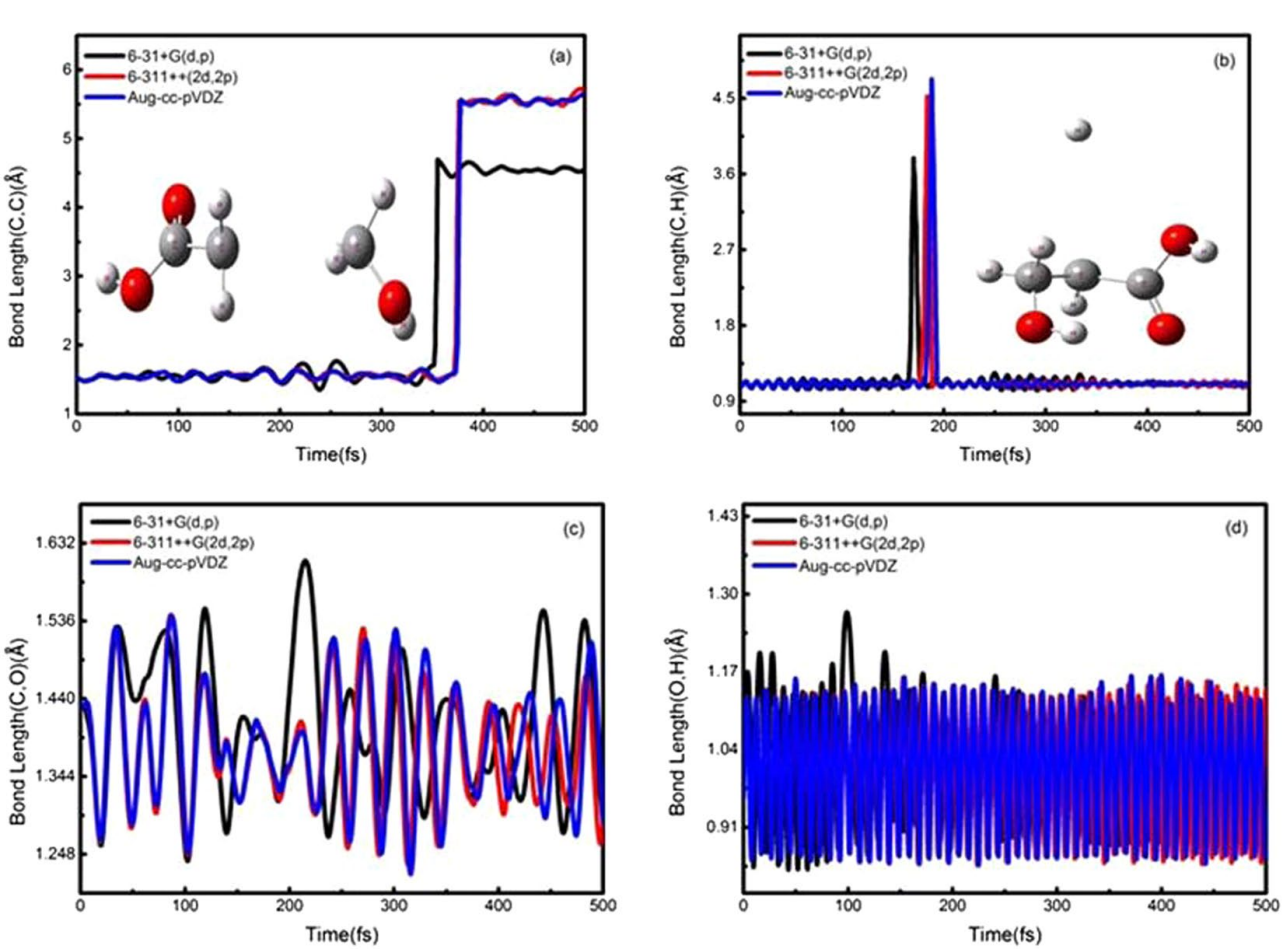
Selected bond distances as a function of the evolution of time in the ADMP simulation process for the isomer: (**a**) C-C bond, (**b**) C-H bond, (**c**) C-O bond, and (**d**) O-H bond. The different colors of trajectories represent the calculation results of different basis sets.

The typical snapshots in Figs. 2 and 3 are chosen on four time points (0,150,200 and 500 fs) to display lactic acid and isomer dissociation process in the ADMP simulations. Figures 4(a) and 5(a) plot the simulated trajectories of lactic acid and isomer, exhibited by the variation in the C-C bond as a function of evolution of time. In Figs. 4(a) and 5(a), the different colors of simulated trajectories correspond to three basis sets. Comparing the C-C bond dissociation trajectories of lactic acid and isomer with the 6-31 + G(d,p), 6-311++G(2d,2p), and Aug-cc-pVDZ basis sets, several interesting points rapidly emerge. First, the C-C bond stretches rapidly after additional cycles of vibration, and the C-C distance elongates linearly with the evolution of time, indicating the direct cleavage of the C-C bond; there is not backed into a bonded state in ADMP trajectory simulation process. Second, the 6-31 + G(d,p) basis set result shows significant differences in dissociation time and trajectory simulation. The present simulation results reveal that the C-C bond of lactic acid and isomer dissociation time and dissociation processes are similar with the 6-311++G(2d,2p) and Aug-cc-pVDZ basis sets.

Figures 4(b) and 5(b) illustrate the simulated time evolution of the C-H bond with the ADMP trajectories corresponding to those of lactic acid and isomer. For lactic acid and its isomer, the C-H bond distance stretches rapidly around 170 fs, and as time proceeds, the C-H bond starts to cleavage. The distant H atom is ~4 Å away from the C atom. Then, the H atom gradually approaches the C atom until the former reaches a bonded state. Given the difference in size of the three basis sets, the C-H bond between the dissociation time and simulated trajectories shows significant differences. However, the C-H bond dissociation trajectories of lactic acid and its isomer with the 6-311++G(2d,2p) and Aug-cc-pVDZ basis set behave similarly, yielding values of 177.3 and 183.8; and176.5 and 182.1 fs, respectively.

The different line colors in Fig. 4(c) indicate the C-O bond dissociation trajectory of lactic acid with the three basis sets. For the 6-311++G(2d,2p) basis set, the C-O bond distance extends around 110 fs until 152.8 fs, whereas the C-O bond is broken. Owing to the electrostatic interactions, the O atom lies near the C atom and sharply returns to a bonded state during the remainder of the dissociation simulation. We compare the three basis sets behavior in terms of the time evolution of the C-O bond distance. The obtained results of the 6-31 + G(d,p) basis set shows an evident difference in dissociation time compared with those of the Aug-cc-pVDZ basis set, where the C-O bond dissociation time is 160.8 fs in the latter case. The difference in the three basis sets agrees with that observed in previous ADMP studies of the DEA dissociation process for small biomolecules^32^. For the 6-311++G(2d,2p) and Aug-cc-pVDZ basis sets, as the basis set size increases, we observe a closer agreement on the dissociation time and trajectory simulation. For the isomer, however, the C-O bond is not broken in the ADMP simulation process, as shown in Fig. 5(c). The trajectory simulation results indicate that the oscillated curve is more complex, similar to the O-H bond, and that vibration mode of the C-O bond produces an effect on its dissociation in the trajectory simulation process. The result with 6-311++G(2d,2p) basis set is highly similar with that of the Aug-cc-pVDZ basis set.

The simulated trajectories of lactic acid of the O-H bond as a function of time evolution are shown by the black, red, and blue lines in Fig. 4(d). Our simulation results for the three basis sets show that the O-H bond is not cleaved in the ADMP simulation for the DEA dissociation process. The simulated trajectories of the O-H bond rapidly present an oscillated curve. Here, the present results suggest that the wagging vibration mode of the O-H bond may affect its dissociation in the DEA dissociation process. R. Janečková *et al*.^33^ have proposed a similar mechanism to explain how the O-H bond vibration mode influences the DEA dissociation process of small biomolecules. The vibrational mode is observed in the DEA of small biomolecules^34^; this mode also plays an important role in the dissociation process. Furthermore, a similar trend is observed when the O-H bond results from the isomer simulated with the three basis sets as presented in Fig. 5(d). For the isomer, the O-H bond is not cleaved in the ADMP simulation for DEA dissociation process. The 6-31 + G(d,p) basis set shows a large discrepancy between the 6-311++G(2d,2p) and Aug-cc-pVDZ basis sets for the O-H bond result of the lactic acid and its isomer.

According to the simulation results, the three basis sets show evident distinction in simulating the dissociation trajectory for lactic acid and its isomer. Especially, the result of the 6-31 + G(d,p) basis set is the most different with the other basis sets. Recently, an assessment theoretical research had shown that the 6-31G + (d,p) basis set had obtained results that agreed with experimental and theoretical investigations on ADMP simulation^22^. However, the 6-31G + (d,p) basis set without polarization functions is unsuited for lactic acid and isomer during ADMP simulation. The Aug-cc-pVDZ basis set is a correlation-consistent basis set, which improves the accuracy but increases computational time. For this reason, the 6-311G++(2d,2p) basis set is the most appropriate and used for ADMP dynamics trajectory simulation of the DEA dissociation process. Our ADMP dynamical simulation results indicate that the C-C, C-H, and C-O bond dissociations of lactic acid and its isomer are the most probable DEA dissociation processes, whose animated dissociation trajectory is used as reference for the lactic acid DEA experimental research. The ADMP dynamical simulation yields accurate and highly efficient computational method of researching molecular dissociation process of DEA.

**Figure 6:**
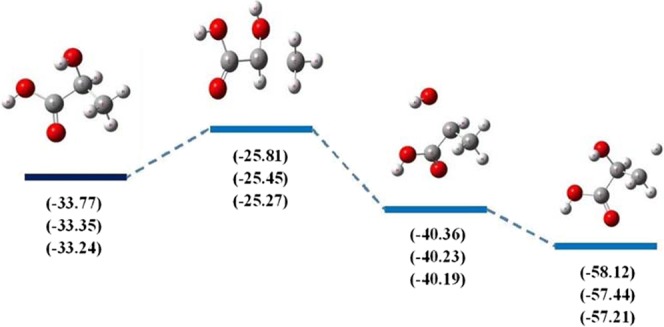
Gibbs Free-energy diagram (kcal mol^−1^) along to dissociation processes of lactic acid obtained with the 6-31 + G(d,p), 6-311++G(2d,2p) and Aug-cc-pVDZ basis set.

**Figure 7:**
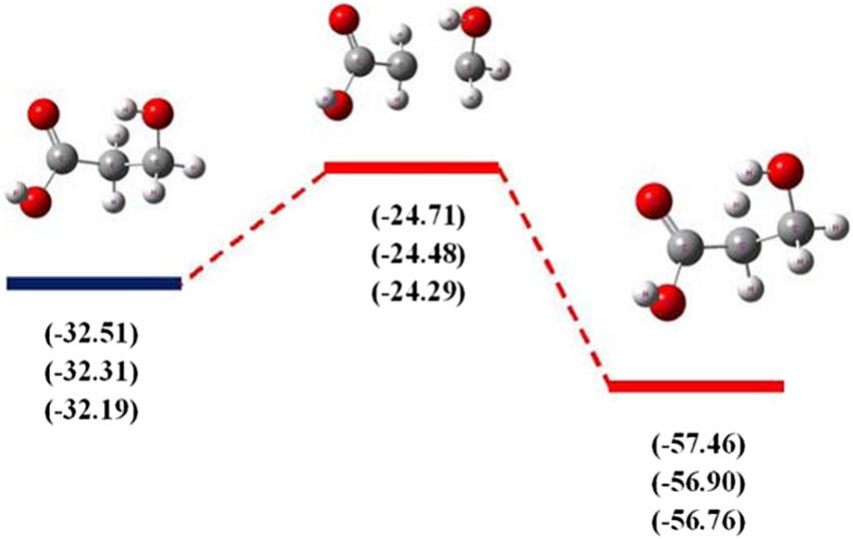
Gibbs Free-energy diagram (kcal mol^−1^) along to dissociation processes of isomer obtained with the 6-31 + G(d,p), 6-311++G(2d,2p) and Aug-cc-pVDZ basis set.

The Gibbs free-energy calculation is widely used to confirm ADMP dissociation processes of lactic acid and isomer. The Gibbs free-energy along dissociation processes is calculated by the 6-31 + G(d,p), 6-311++G(2d,2p) and Aug-cc-pVDZ basis set, as shown in Figs. 6 and 7, respectively. According to these two figures, the Gibbs free-energy diagrams predict that the C-H bond is the most active in dissociation processes, clearly highlighting the C-H bond is easily broken in dissociation processes from a thermodynamic perspective. For lactic acid, the C-O bond is lower active than the C-H bond in dissociation processes, which the values of Gibbs free-energy are −40.36, −40.23, −40.19 kcal mol^−1^ with respect to the 6-31 + G(d,p), 6-311++G(2d,2p) and Aug-cc-pVDZ basis set. The highest values of Gibbs free-energy are obtained from dissociation processes of the C-C bond for lactic acid and isomer. This suggests that the C-C bond is broken after the C-H bond cleavage.

### NBO analysis of the dissociation process of lactic acid and its isomer

**Figure 8:**
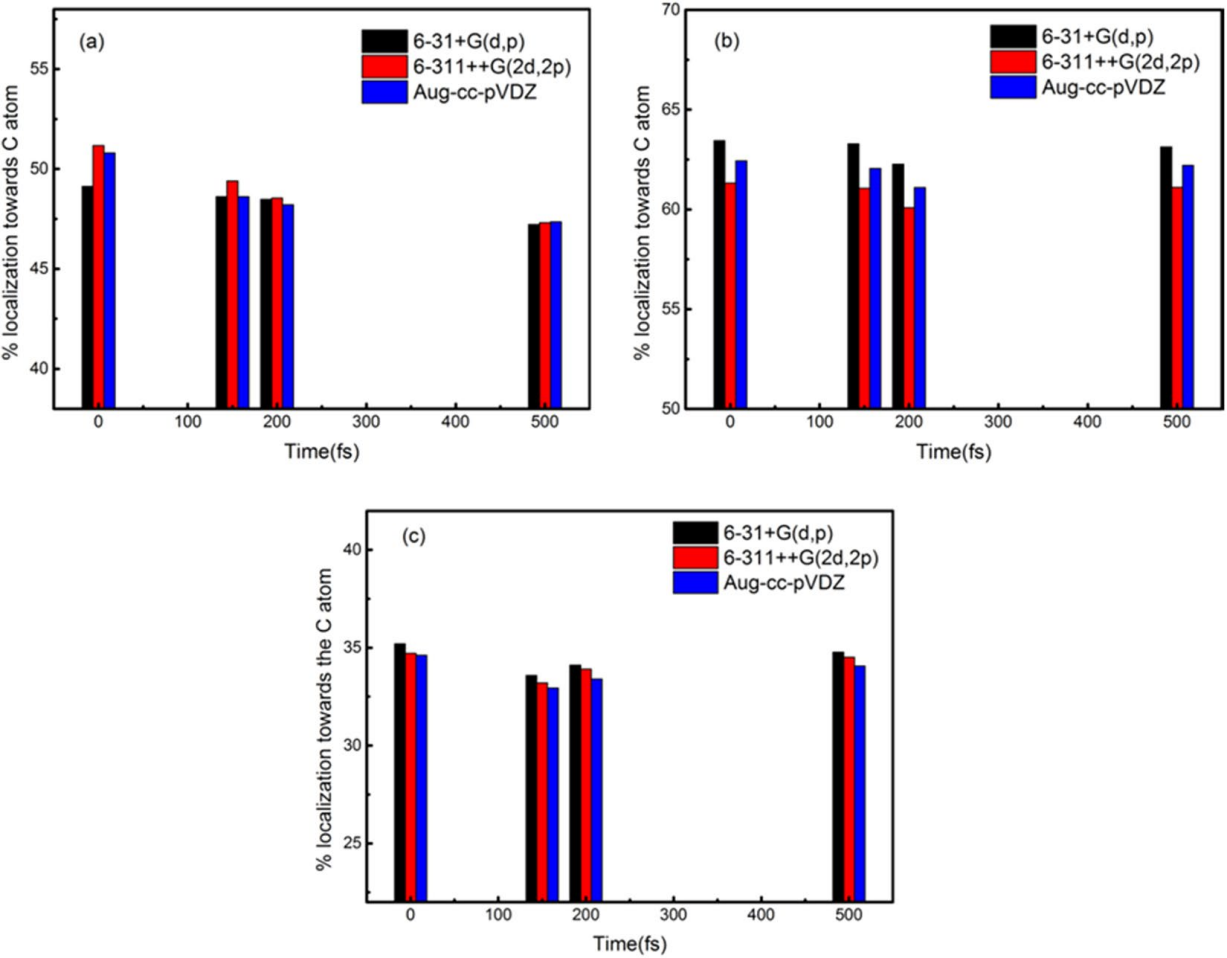
NBO analysis of the variations in the C-C (**a**), C-H (**b**), and C-O (**c**) bond localization toward the C atom along the ADMP simulation from lactic acid.

**Figure 9:**
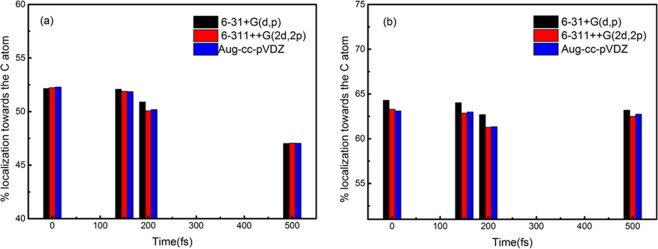
NBO analysis of the variations in the C-C (**a**) and C-H (**b**) bond localization toward the C atom in the ADMP simulation for the isomer.

The calculation of the NBO method is the most prevalent method of charge population and charge transfer analysis, which are the foremost applications of quantum chemistry calculation in biomolecule systems. Figures 8 and 9 show the charge population data calculated with NBO analysis during the ADMP simulations of lactic acid and isomer dissociation. Notably, the results of charge population are distinct for the three basis sets for lactic acid and its isomer, suggesting that the variations in charge population are accompanied by the C-C, C-H, and C-O bond dissociation processes.

At the beginning of simulation for Fig. 8(a), the charge populations of the C-C bond reach 49.13%, 51.17%, and 50.8% localization toward the C atom from the 6-31G + (d,p), 6-311++G(2d,2p), and Aug-cc-pVDZ basis sets, respectively. Around 370 fs, the dissociation process is short-lived, the C-C bond is cleaved, and the charge population is changed, whereas the values of charge population of the C-C bond localization toward the C atom are the lowest. The trend of charge population rises again, until the charge population of the C-C bond approaches 47.22%, 47.31%, and 47.35% localization toward the C atom, which are little equivalent to the prime simulation with the 6-31G + (d,p), 6-311++G(2d,2p), and Aug-CC-pVDZ basis sets, respectively. Similar results can be found for the C-H bond localization toward the C atom in Fig. 8(b). Around 170 fs, the C-H bond cleaved, and the charge population changes, whereas the values of the C-H bond localization toward the C atom total 63.44%, 61.32%, and 62.43% with the 6-31G + (d,p), 6-311++G(2d,2p), and Aug-CC-pVDZ basis sets, respectively. At 500 fs, the values of the C-H bond localization toward the C atom reach 62.75%, 61.12%, and 61.91%, which are less than the prime simulation with the 6-31 + G(d,p), 6-311++G(2d,2p), and Aug-CC-pVDZ basis sets, respectively. Figure 8(c) depicts the C-O bond charge population localization toward the C atom with the three basis sets for lactic acid. As shown in Fig. 8(c), the charge population evidently decreases to the simulation time of 150 fs, and the values of the C-O bond localization toward the C atom amount to 35.2%, 34.72%, and 34.62% with the 6-31G + (d,p), 6-311++G(2d,2p), and Aug-CC-pVDZ basis sets, respectively. Remarkably, the difference in charge population of the three basis sets is accompanied by the C-O bond dissociation process. A similar research on biomolecules has previously been demonstrated^14^.

For the isomer in Fig. 9(a), the C-C bond charge population localization toward the C atom values reach 52.15%, 52.23%, and 52.28% with the 6-31 + G(d,p), 6-311++G(2d,2p), and Aug-cc-pVDZ basis sets, respectively, for the initial ADMP simulations. The most important variation is observed for the C-C bond cleavage, whose charge population localization toward the C atom reaches 47.02%, 47.05%, and 47.04% with the 6-31 + G(d,p), 6-311++G(2D,2P), and Aug-cc-pVDZ basis sets, respectively. These results indicate that any variations in charge population are affected by the chemical bond changes between lactic acid and its isomer. Figure 9(b) illustrates the C-H bond charge population localization toward the C atom of isomer; the variations trend is similar with that of lactic acid.

Comparing the results of NBO analysis charge population for lactic acid and its isomer, differences in the three studied basis sets, including the influence of charge population along with ADMP dissociation processes, are notable, especially when using the 6-311++G(2d,2p) and Aug-cc-pVDZ basis sets. Our calculations indicate that the use of NBO analysis charge population is reasonable with large basis sets, such as 6-311++G(2d,2p) and Aug-cc-pVDZ. From the results of previous research, the charge population of NBO analysis may be a better candidate for larger basis sets with more diffuse functions^13^. Considering the size of this research system and computational time, the 6-311++G(2d, 2p) basis set is the best choice for charge population of NBO analysis.

Tables 4–6 present the results of second-order perturbation theory analysis of the NBO Fock Matrix at the B3LYP/6-31 + G(d,p), 6-311++G(2d,2p), and Aug-cc-pVDZ levels for lactic acid and its isomer. Second-order perturbation theory analysis, which evaluates charge transfer of the donor−acceptor, can help us to understand the C-C, C-H, and C-O bond stabilization in the DEA dissociation processes for lactic acid and its isomer. The tables summarize the charge transfer and stabilization energy of the selected C-C, C-H, and C-O bonds along with lactic acid and isomer dissociation process. Table 4 shows that the charge transfer of lactic acid and its isomer are obtained from BD(1)C1-C5 and BD(1)C1-C8 orbitals into BD*(1)C7-O9, BD*(1)C5-H6,BD*(2)C7-O8, BD*(1)C1-H3, BD*(2)C4-O5, and BD*(1)C4-O6 anti-bonding orbitals with the 6-31 + G(d,p), 6-311++G(2d,2p), and Aug-cc-pVDZ basis sets. As the charge transfer accompanies the dissociation of the C-C, C-H, and C-O bonds and plays a major role in this process, the stabilization energy E(2) can be used as an index to assess the cleavage of the C-C, C-H, and C-O bonds. For lactic acid, charge transfer occurs from BD(1)C1-C5 orbital into BD*(1)C5-H6 anti-bonding orbitals, and the stabilization energy E(2) values are the lowest (0.68 kcal mol^−1^) with the 6-31 + G(d,p) basis set, followed by the Aug-cc-pVDZ (0.75 kcal mol^−1^) and 6-311++G(2d,2p)(0.78 kcal mol^−1^) basis sets. A high stabilization energy E(2) from BD(1)C1-C5 orbital into BD*(1)C7-O9 anti-bonding orbital is observed using the 6-311++G(2d,2p) and Aug-cc-pVDZ basis sets, with values of 3.57 and 3.73 kcal mol^−1^, respectively. For convenient discussion, the charge transfer of isomer in Table 4 can be given as follows: BD(1)C1-C8→BD*(1)C1-H3, BD(1)C1-C8→BD*(2)C4-O5, and BD(1)C1-C8→BD*BD*(1)C4-O6. As for the stabilization energy E(2) of the BD(1)C1-C8→BD*(1)C1-H3, the value with 6-31 + G(d,p) basis set is weaker than that of the 6-311++G(2d,2p) and Aug-cc-pVDZ basis sets. Table 5 presents the calculated charge transfer and stabilization energy of the C-H bond of lactic acid and its isomer. According to the values of stabilization energy E(2) listed in Table 5, a charge transfer occurs from BD(1)C1-H2 orbital into BD*(1)C5-O11, BD*(2)C4-O5, and BD*(1)C8-H9 anti-bonding orbitals, providing the weakest stabilization with 6-31 + G(d,p) basis set. The values of stabilization energy E(2) with 6-311 + G(2d,2p) basis set are similar to that of the Aug-cc-pVDZ basis set. Table 5 summarizes the lactic acid stabilization energy and charge transfer from BD(1)C5-O11 orbital into BD*(1)C1-H2 and BD*(2)C7-O8 anti-bonding orbitals. From Table 6, the values of stabilization energy E(2) are less than 2 kcal mol^−1^ with the three basis sets, suggesting that the C–O bond exhibits a weak stabilization accompanied by charge transfer. The obtained stabilization energy of lactic acid and its isomer with the three basis sets are generally low, indicating that the C-C, C-H, and C-O bonds are prone to cleavage. In comparison of Tables 4–6, the calculated stabilization energy E(2) results of the C-C, C-H, and C-O bonds with the 6-311++G(2d,2p) and Aug-cc-pVDZ basis sets are higher than those obtained with the 6-31 + (d,p) basis set for the lactic acid and its isomer. The above NBO analysis results with the three basis sets provide insights into the C-C, C-H, and C-O bond cleavage in the DEA dissociation processes from a different perspective.

**Table 4:** NBOs with second-order perturbation stabilization energy E(2) (kcal mol^−1^) analysis of the C-C bond for lactic acid and its isomer. Results are obtained at the B3LYP/6-31 + G(d,p), 6-311++G(2d,2p), and Aug-cc-pVDZ levels.

	Method	Donor	Acceptor	E(2)
Lactic acid	B3LYP/6-31 + G(d,p)	BD(1)C1-C5	BD*(1)C7-O9	3.47
		BD(1)C1-C5	BD*(1)C5-H6	0.68
		BD(1)C1-C5	BD*(2)C7-O8	0.96
	B3LYP/6-311++G(2d,2p)	BD(1)C1-C5	BD*(1)C7-O9	3.57
		BD(1)C1-C5	BD*(1)C5-H6	0.78
		BD(1)C1-C5	BD*(2)C7-O8	1.13
	B3LYP/Aug-cc-pVDZ	BD(1)C1-C5	BD*(1)C7-O9	3.73
		BD(1)C1-C5	BD*(1)C5-H6	0.75
		BD(1)C1-C5	BD*(2)C7-O8	0.97
Isomer	B3LYP/6-31 + G(d,p)	BD(1)C1-C8	BD*(1)C1-H3	0.62
		BD(1)C1-C8	BD*(2)C4-O5	4.12
		BD(1)C1-C8	BD*(1)C4-O6	0.57
	B3LYP/6-311 ++ G(2d,2p)	BD(1)C1-C8	BD*(1)C1-H3	0.63
		BD(1)C1-C8	BD*(2)C4-O5	4.17
		BD(1)C1-C8	BD*(1)C4-O6	0.65
	B3LYP/Aug-cc-pVDZ	BD(1)C1-C8	BD*(1)C1-H4	0.70
		BD(1)C1-C8	BD*(2)C4-O5	4.23
		BD(1)C1-C8	BD*(1)C4-O6	0.69

**Table 5:** NBOs with second-order perturbation stabilization energy E(2) (kcal mol^−1^) analysis of the C-H bond for lactic acid and its isomer. Results are obtained at the B3LYP/6-31 + G(d,p), 6-311++G(2d,2p), and Aug-cc-pVDZ levels.

	Method	Donor	Acceptor	E(2)
Lactic acid	B3LYP/6-31 + G(d,p)	BD(1)C1-H2	BD*(1)C5-O11	3.9
	B3LYP/6-311++G(2d,2p)	BD(1)C1-H2	BD*(1)C5-O11	4.36
	B3LYP/Aug-cc-pVDZ	BD(1)C1-H2	BD*(1)C5-O11	5.06
Isomer	B3LYP/6-31 + G(d,p)	BD(1)C1-H2	BD*(2)C4-O5	6.03
		BD(1)C1-H2	BD*(1)C8-H9	2.35
	B3LYP/6-311++G(2d,2p)	BD(1)C1-H2	BD*(2)C4-O5	6.32
		BD(1)C1-H2	BD*(1)C8-H9	2.32
	B3LYP/Aug-cc-pVDZ	BD(1)C1-H2	BD*(2)C4-O5	6.15
		BD(1)C1-H2	BD*(1)C8-H9	2.28

**Table 6:** NBOs with second-order perturbation stabilization energy E(2) (kcal mol^−1^) analysis of the C-O bond for lactic acid. Results are obtained at the B3LYP/6-31 + G(d,p), 6-311++G(2d,2p), and Aug-cc-pVDZ levels.

	Method	Donor	Acceptor	E(2)
Lactic acid	B3LYP/6-31 + G(d,p)	BD(1)C5-O11	BD*(1)C1-H2	1.01
		BD(1)C5-O11	BD*(2)C7-O8	1.51
	B3LYP/6-311++G(2d,2p)	BD(1)C5-O11	BD*(1)C1-H2	1.11
		BD(1)C5-O11	BD*(2)C7-O8	1.53
	B3LYP/Aug-cc-pVDZ	BD(1)C5-O11	BD*(1)C1-H2	1.05
		BD(1)C5-O11	BD*(2)C7-O8	1.62

## Conclusions

In the present paper, the dissociation dynamics of lactic acid and its isomer in gas phase for the DEA process is investigated by using *ab* initio MD simulations combined ADMP method. The ADMP simulations coupled with the B3LYP theory of the 6-31G + (d,p), 6-311++G(2d,2p), and Aug-cc-pDVZ basis sets are employed to optimize the ground state geometries and obtain dipole moments and harmonic vibrational frequencies of lactic acid and its isomer. The ADMP dynamics trajectory simulation results indicate that the C-C, C-O, and C-H bond result from the cleavage of lactic acid and its isomer within femtoseconds of the simulation time scale in the DEA dissociation process, and the difference in dissociation trajectories depends on the size of the three basis sets and vibration mode of the chemical bond. ADMP simulation is a qualitative analysis of the dynamic dissociation trajectory in the DEA process for lactic acid and its isomer, and it can be helpful in performing experimental research. The analysis of NBO calculation results of charge population and second-order perturbation theory for lactic acid and its isomer indicate the charge transfer between the C-C, C-O, and C-H bonds, and this result confirms dissociation in the DEA process from a different perspective. Comparison between the ADMP and NBO analysis results reveal that the 6-311++G(2d,2p) is an excellent basis set for lactic acid and isomer theoretical calculations of the dissociation dynamics of DEA processes.

On-going works from our group aim at the DEA experiments of lactic acid, in light of the conclusions drawn from this study. Finally, we believe that the research on lactic acid and its isomer and dissociation dynamics of DEA can be useful for understanding the radiation damage with biological systems, in particular, to guide scientists in conducting lactic acid DEA experiments in the future.
